# Psychometric properties of the TMMS-24 emotional intelligence scale in Peruvian university students

**DOI:** 10.3389/fpsyg.2025.1611923

**Published:** 2025-06-09

**Authors:** Pablo Fernández-Berrocal, Vilma Vilca-Pareja, Maria Elena Rojas Zegarra, Manuel Edmundo Hillpa-Zuñiga, Victor Ritchar Yana-Calla, Rosario Cabello

**Affiliations:** ^1^Department of Basic Psychology, Faculty of Psychology, University of Málaga, Málaga, Spain; ^2^Escuela de Posgrado de la Universidad Católica de Santa María, Arequipa, Peru; ^3^Escuela Profesional de Psicología de la Universidad Nacional de San Agustín de Arequipa, Arequipa, Peru; ^4^Escuela Profesional de Ingeniería Comercial, Facultad de Ciencias Económico Administrativas, Universidad Católica de Santa María, Arequipa, Peru; ^5^Department of Developmental and Educational Psychology, University of Málaga, Málaga, Spain

**Keywords:** emotional intelligence, emotional attention, emotional clarity, emotional repair, psychometric properties, Peruvian university students

## Abstract

**Introduction:**

This study investigates the psychometric properties of the Spanish-adapted version of the Trait Meta-Mood Scale (TMMS-24) in Peruvian university students. University life presents significant challenges that can negatively impact students’ mental health, increasing the prevalence of anxiety and depression. Emotional intelligence (EI) has been identified as a crucial protective factor in this context. The TMMS-24 is a widely used self-report instrument that assesses individuals’ perceptions of their own EI, encompassing three dimensions: emotional attention, emotional clarity, and emotional regulation.

**Method:**

This study analyzed the psychometric properties pertaining to TMMS-24, such as the reliability and validity of this instrument on Peruvian Students. The analysis was made on 1315 students whose ages ranged from 18 to 30 years of age (M = 20.03, SD = 2.24).

**Results:**

The findings confirmed high reliability and internal consistency, with Cronbach’s alpha coefficients exceeding 0.80 for all three subscales. Test-retest reliability, a novel finding in the Peruvian context, was significant, indicating good temporal stability. Confirmatory Factor Analysis supported the original three-dimensional structure (Attention, Clarity, and Repair). Construct validity was evidenced by factor loadings ranging from 0.32 to 0.85, which is consistent with previous research. Regarding gender differences, males reported significantly higher scores in emotional clarity and repair, while no significant differences were found in emotional attention. A positive correlation between cognitive reappraisal and EI and a negative correlation between suppression and EI supported convergent and discriminant validity. Furthermore, significant positive correlations were observed between all TMMS-24 dimensions and personality traits (Extraversion, Agreeableness, Conscientiousness, Neuroticism, and Openness). These results provide evidence for the reliability and validity of the TMMS-24 for assessing perceived EI in Peruvian university students.

**Discussion:**

These findings have significant implications for researchers and educational interventions in Peru and their impact on mental health and academic success in this population.

## Introduction

University life represents a significant challenge for young people, as it can affect their mental health and increase the incidence of anxiety, stress, or depression (American College Health Association, 2021; Cao et al., 2020; Wang et al., 2021; Wathelet et al., 2020). In this context, most students are in adolescence or early adulthood and must cope with various risk factors, such as academic pressure, financial difficulties, romantic breakups, academic failure, and separation from their family environment (Debowska et al., 2022). Moreover, recent studies have shown an increase in anxiety and depression symptoms compared to the period before the pandemic (Lacomba-Trejo et al., 2024; Sánchez-López et al., 2024). A key protective factor against these challenges is the development of emotional intelligence (EI) (Delgado et al., 2024; Koçak, 2021).

EI is defined as the ability to perceive, understand, and regulate both one’s own emotions and those of others (Mayer and Salovey, 1997; Mayer et al., 2016; Salovey and Mayer, 1990). Its study is approached from different theoretical perspectives. Joseph and Newman (2010) identified three main models:

1. Ability model, which views EI as a form of intelligence focused on processing emotional information. It is assessed through performance-based tests, such as the Mayer-Salovey-Caruso Emotional Intelligence Test (MSCEIT) (Mayer et al., 2002).
2. Self-report ability model, where individuals assess their own EI through scales such as the Trait Meta-Mood Scale (TMMS) (Salovey et al., 1995).
3. Mixed self-report model, which conceives EI as a personality trait, integrating interpersonal and intrapersonal skills. A widely used instrument in this approach is the Trait Emotional Intelligence Questionnaire (TEIQue) (Petrides et al., 2007).

Several studies have demonstrated the protective role of EI in mental health and subjective well-being. For example, Mancini et al. (2024) conducted a meta-analysis showing that higher EI is associated with lower levels of anxiety and depression, greater emotional stability, more effective coping strategies, better interpersonal relationships, and greater socio-emotional competence. Similarly, Llamas-Díaz et al. (2022), in another meta-analysis, found a significant positive relationship between EI and subjective well-being in adolescents. Furthermore, numerous studies support the relationship between EI and general well-being (Delhom et al., 2017; Hidalgo-Fuentes et al., 2021; Martín-Talavera et al., 2024; Mikolajczak et al., 2008; Sánchez-Álvarez et al., 2016; Shengyao et al., 2024; Torrelles-Nadal et al., 2024; Vega et al., 2022; Zeidner et al., 2009), as well as with physical health (Martins et al., 2010) and mental health (Barros and Sacau-Fontenla, 2021; Domínguez-García and Fernández-Berrocal, 2018; Dongmei, 2024; Lacomba-Trejo et al., 2024; Salguero et al., 2012; Schutte et al., 2007; Shen et al., 2021). One of the most widely used instruments to assess EI is the Trait Meta-Mood Scale (TMMS), which measures individuals’ self-perceived emotional intelligence without right or wrong answers (Salovey et al., 1995). The Spanish-adapted version of the TMMS consists of 24 items grouped into three dimensions (Fernández-Berrocal et al., 2004): (1) Emotional Attention, which reflects the degree to which individuals believe they pay attention to their emotions; (2) Emotional Clarity, which indicates the perceived ability to identify and understand one’s own emotional states; and (3) Emotional Repair or Regulation, which measures the ability to reduce negative emotions and maintain positive emotional states (Salovey and Mayer, 1990). The study by Fernández-Berrocal et al. (2004) developed a reduced version. The TMMS-24, while maintaining the original scale’s three-factor structure. The revision was primarily motivated by the low reliability observed in the Spanish version of the instrument. Items were eliminated based on their low contribution to reliability and through semantic analysis. Furthermore, negative item wording was changed to a positive format to improve comprehension within the Spanish population. Results with university students showed high reliability for each component (Cronbach’s *α* = 0.90, 0.90, and 0.86 for Emotional Attention, Emotional Clarity, and Emotional Repair, respectively) and evidence of temporal stability for a 4-week interval (test–retest correlations = 0.60, 0.70, and 0.83, respectively).

The TMMS-24 has been validated in various countries and populations, demonstrating high reliability and psychometric validity in both adults and adolescents (Fernández-Berrocal et al., 2004; Martín-Albo et al., 2010; Ondé et al., 2021; Patti-Signorelli and de Romero-Díaz, 2023; Salguero et al., 2010; Valdivia et al., 2015). In Spain, Delhom et al. (2017) validated the three-factor structure in older adults, and Ondé et al. (2021) confirmed its reliability and multidimensional nature. Górriz et al. (2021) in a cross-cultural study conducted in Argentina, Ecuador, and Spain, it was concluded that the scale shows convergent and discriminant validity. Similarly, Gonzalez et al. (2021) found that the TMMS-24 is a psychometrically valid tool for assessing emotional intelligence in Argentine university students. In Mexico, Valdivia et al. (2015) evaluated its factorial structure through confirmatory factor analysis, finding adequate model fit. In Brazil, Câmara et al. (2023) found that the TMMS-24 is psychometrically suitable for assessing EI among adolescents. In Poland, Cabello et al. (2025) confirmed its internal consistency and three-dimensional structure in both the original and the Spanish-adapted versions. In Italy, Patti-Signorelli and de Romero-Díaz (2023) confirmed the stability of its psychometric properties in comparison with both the original version by Salovey and its Spanish adaptation (Fernández-Berrocal et al., 2004).

Additionally, the TMMS-24 has shown significant relationships with other psychological constructs, such as personality and emotional regulation. Saklofske et al. (2007) found correlations between self-reported EI and personality traits. Antoñanzas et al. (2014) evidenced its relationship with extraversion, while Ghiabi and Besharat (2011) identified associations with the five major personality dimensions. Mayer et al. (2004) found correlations between EI and traits such as agreeableness, openness to experience, and conscientiousness. Likewise, Lopes et al. (2003) linked emotional management to extraversion. On the other hand, Pastor et al. (2019) showed that cognitive reappraisal is associated with emotional attention and regulation, whereas expressive suppression is negatively related to emotional clarity and regulation in the TMMS-24. Despite extensive evidence supporting the validity of the TMMS-24 in various contexts, its study remains limited in Latin America, and particularly in Peru. Research in Peru has been conducted with small samples (Bueno-Cuadra et al., 2023) and has not deeply explored its psychometric properties (Bueno-Cuadra et al., 2023; Pérez-Zárate et al., 2021; Ruiz et al., 2022). Specifically, Ruiz et al. (2022) highlighted the need to assess factorial invariance as well as predictive and concurrent validity. Likewise, Pérez-Zárate et al. (2021) suggested a more detailed analysis of its factorial structure.

The urgency to validate the TMMS-24 scale on Peruvian university students is found to be imperative to expand socio-cultural analysis particularities that shape self-assessment and perception of emotional intelligence (EI) in this specific context. EI, which is understood to be a psychological construct is highly influenced by contextual factors and as such, cannot be considered as universal or homogenous in itself. Much to the contrary, it is regulated by moral systems, emotional norms, and social practices that are unique within each culture (Mikolajczak et al., 2008; Cabello et al., 2025). When it comes to Peruvians within university settings, it is distinguished by a structural heterogeneity from being subject to socio-economic limitation, ethno-linguistic diversity, and finite institutionalized mental health politics. Conditions which directly impact the application, development, and recognition of emotional competences (Barros and Sacau-Fontenla, 2021; Bueno-Cuadra et al., 2023).

In this regard, multiple studies have highlighted the need to authenticate and adapt emotional intelligence (EI) instruments catering to cultural and psychosocial specificities of the context by taking into consideration that that emotional regulation strategies and coping mechanisms are culturally determined constructs rather than universal ones (Fernández-Berrocal et al., 2004; Zeidner et al., 2009). By incorporating a contextualized approach, not only will the finding be provided with greater interpretive accuracy, but it will also strengthen the relevance and legitimacy of the TMMS-24 as a diagnostic tool in Latin American contexts where empirical development on EI is still in development (Pérez-Zárate et al., 2021; Mancini et al., 2024).

In this context, the present study aimed to analyze the psychometric properties of the TMMS-24 in Peruvian university students. Specifically, it sought to:

- Assess the reliability and internal consistency of the TMMS-24.
- Analyze construct validity through confirmatory factor analysis (CFA) and correlations among its three subscales.
- Examine gender differences in EI as measured by the TMMS-24.
- Evaluate discriminant and convergent validity by correlating the TMMS-24 with personality traits and emotional regulation.

## Method

### Participants

The study was conducted at two universities, one public and one private, in Arequipa, Peru. A total of 1,315 students participated. A non-probabilistic intentional sampling method was used. Participants’ ages ranged from 18 to 30 years (*M* = 20.03, SD = 2.24); 863 (65.6%) were female and 452 (34.4%) were male. The criteria for inclusion were: to be a regular university student (belonging to either a public or private institution), to be within at least the 18 to 30-year-old age bracket, and to provide written consent.

### Instruments

#### Trait meta-mood scale TMMS-24

This instrument was developed by Salovey et al. (1995) and measures emotional attention, clarity, and repair. The original version consists of 48 items. In this study, the brief version adapted to Spanish by Fernández-Berrocal et al. (2004) was used. It includes 24 items grouped into three dimensions: (1) emotional attention (Item example; I pay attention to my feelings) (2) emotional clarity (Item example; I am aware of my feelings), and (3) emotional repair (Item example; I try to stay positive despite being in an emotionally compromised state), with 8 items per dimension. The scale uses a 5-point Likert format (1 = strongly disagree to 5 = strongly agree).

#### Emotion regulation questionnaire (ERQ)

This questionnaire was developed by Gross and John (2003) and consists of two dimensions: (1) cognitive reappraisal, a strategy for regulating emotions, and (2) expressive suppression, which involves modulating the emotional response in progress. The scale has 10 items—six measuring cognitive reappraisal and four measuring expressive suppression. It uses a 7-point Likert scale (1 = strongly disagree to 7 = strongly agree). In Peru, it was validated by Gargurevich and Matos (2010), who examined the instrument’s psychometric properties in 320 students from private universities. They confirmed construct validity and found the instrument to have adequate reliability, with Cronbach’s alpha coefficients above 0.70 for both dimensions.

#### Mini-international personality item pool (Mini-IPIP)

This instrument is based on the Five-Factor Theory of personality and was originally proposed by Goldberg (1999) with a 50-item version. The short version, reduced to 20 items, was developed by Donnellan et al. (2006), and includes five dimensions: extraversion, agreeableness, conscientiousness, neuroticism, and openness. Items are rated on a 5-point Likert scale (1 = strongly disagree to 5 = strongly agree). In Peru, Yupanqui-Lorenzo et al. (2021) validated the instrument in a sample of 521 university students of both sexes in Lima. They confirmed its internal structure and reliability, obtaining omega coefficients greater than 0.70 for each factor.

### Procedure

This research was approved by the Ethics Committee of the Universidad Católica de Santa María before its execution. The instruments were administered virtually through the TEAMS platform. The obtained data were coded in Excel 360, and a database was then created and exported to RStudio for further analysis. A test–retest of the TMMS-24 was conducted with 208 out of the 1,315 participants; a code was assigned to identify them. The retest was administered 2 weeks later.

## Results

### Descriptive item statistics

Descriptive statistics were calculated by; mean, standard deviation, skewness, and kurtosis, as proposed by Ferrando et al. (2022) and Muñiz (2018). Table 1 presents the descriptive statistics of the items. The item means range from 2.69 to 3.34, and the standard deviations range from 0.96 to 1.1, indicating no floor or ceiling effects. Regarding shape statistics, skewness values range from-0.34 to 0.50, and kurtosis values range from-0.50 to−0.90; these values fall within the recommended thresholds for assuming univariate normal distribution (Ferrando et al., 2022; Hair et al., 2014; Kline, 2016).

**Table 1:** Descriptive statistics of the instrument’s items.

Items	M	(SD)	g_1_	g_2_
Item 01	3.10	(1.05)	0.14	-0.90
Item 02	3.12	(1.07)	0.09	−0.87
Item 03	2.96	(1.10)	0.25	−0.87
Item 04	3.34	(1.07)	−0.04	−0.90
Item 05	2.71	(1.07)	0.50	−0.50
Item 06	2.90	(1.05)	0.22	−0.68
Item 07	2.96	(1.03)	0.25	−0.70
Item 08	3.03	(1.03)	0.22	−0.75
Item 09	2.91	(1.08)	0.18	−0.78
Item 10	2.93	(1.05)	0.15	−0.73
Item 11	2.98	(1.02)	0.15	−0.71
Item 12	3.03	(1.01)	0.12	−0.73
Item 13	3.14	(0.96)	0.12	−0.71
Item 14	2.72	(1.08)	0.33	−0.63
Item 15	2.83	(1.03)	0.30	−0.61
Item 16	2.95	(1.02)	0.15	−0.72
Item 17	3.03	(1.11)	0.06	−0.80
Item 18	3.04	(1.08)	0.05	−0.70
Item 19	2.69	(1.11)	0.28	−0.72
Item 20	2.99	(1.07)	0.15	−0.75
Item 21	3.05	(1.07)	0.03	−0.69
Item 22	3.06	(1.05)	0.12	−0.71
Item 23	3.66	(1.07)	−0.34	−0.77
Item 24	3.06	(1.03)	0.19	−0.70

Min, minimum value; Max, maximum value of the item; M, mean; SD, standard deviation; g_1_, skewness; g_2_, kurtosis.

### Factorial validity

**Figure 1:**
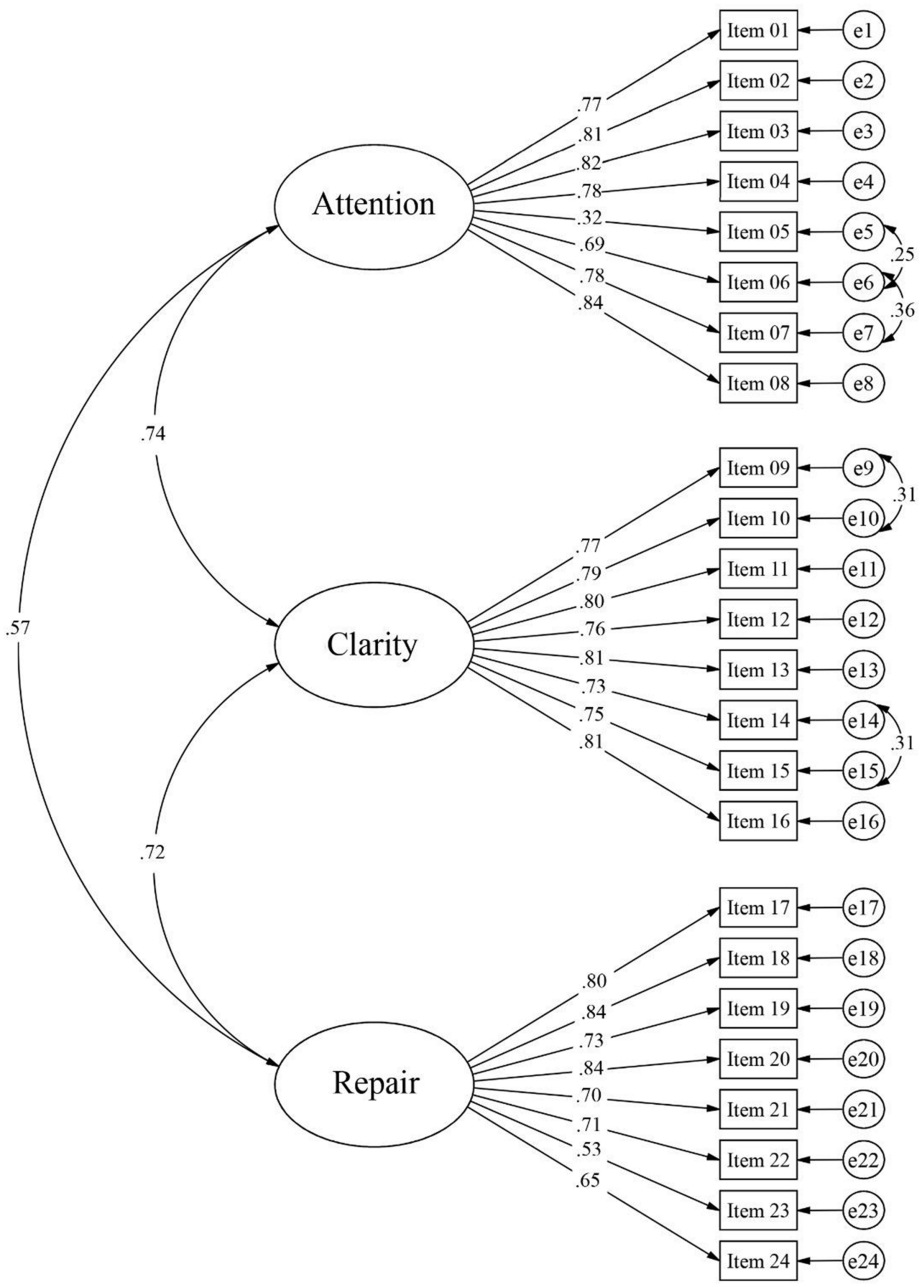
Factor loadings of the TMMS-24 obtained from the CFA.

For the CFA, it is taken into account that items must have at least five response alternatives to be considered continuous (Lloret-Segura et al., 2014), which applies to our instrument. Prior to the factorial analysis, multivariate normal distribution was verified using Mardia’s test (kurtosis = 128.0 with *p* < 0.05), indicating that this assumption was not met. Therefore, the robust maximum likelihood estimator (MLR) was used. The results of the factor loadings can be seen in Figure 1. This model yielded good fit indexes. For this, the following cutoff points are taken into account: Comparative Fit Index (CFI) ≥ 0.95, Tucker-Lewis Index (TLI) ≥ 0.95, Root Mean Square Error of Approximation (RMSEA) ≤ 0.05, and Standardized Root Mean Residual (SRMR) ≤ 0.06 (Keith, 2019). The calculated fit index values for the final model are: *χ*^2^(245) = 853.69, *p* < 0.01; CFI = 0.956; TLI = 0.951; RMSEA = 0.043; and SRMR = 0.049. Factor loadings in this model range from 0.32 to 0.84 (Figure 1).

### Instrument reliability

#### Internal consistency

Table 2 presents the reliability of the instrument. Internal consistency methods were used through Cronbach’s alpha and McDonald’s omega coefficients, obtaining values above 0.70 in the three scales—that is, attention, clarity, and emotional repair. This demonstrates that the dimensions hold acceptable internal consistency according to Tavakol and Dennick (2011) proposal seeing as, the values higher than seven are admissible; the instrument is found to be highly reliable. An AVE (Average Variance Extracted) greater than 0.50 was also obtained.

**Table 2:** Instrument reliability: scale comparison by sex.

Variables	*α*	*ω*	AVE	Women (863)	Men (452)
				M (SD)	M (SD)
Attention	0.898	0.900	0.548	24.14 (6.32)	24.06 (6.90)
Clarity	0.926	0.926	0.610	22.79 (6.63)	24.83 (6.63)
Repair	0.899	0.901	0.534	23.75 (6.52)	26.16 (6.40)

*α*, Cronbach’s alpha coefficient; *ω*, McDonald’s omega; AVE, Average Variance Extracted.

#### Sex comparison and stability test

Temporal stability was assessed in 208 participants, yielding high and significant correlations: for attention, *r*_(test–retest)_ = 0.741, *p* < 0.01; for clarity, *r*_(test–retest)_ = 0.722, *p* < 0.01; and for repair, *r*_(test–retest)_ = 0.721, *p* < 0.01. Regarding sex differences, comparisons were made, and on the clarity scale *t*_(1313)_ = 5.311, *p* < 0.01, *d* = 0.31, and on the repair scale *t*_(1313)_ = 6.397, *p* < 0.01, *d* = 0.37, males obtained higher scores than females, with small effect sizes. In the attention dimension, scores were similar for both sexes.

### Factorial invariance analysis

The factorial invariance of the measurement model across gender identities, specifically, the for male and female participants—was systematically evaluated through a multi-group analysis, adhering to the procedural hierarchy outlined by Chen (2007). This analytical sequence encompassed successive examinations of configural, metric, scalar, and strict invariance, each composing a progressively stringent test of measurement equivalence.

Initially, the configural invariance was probed to verify whether the overarching factorial configuration—encompassing both the dimensionality and the factor-loading architecture—was replicable across the gender-based subsamples. Subsequently, metric invariance was scrutinized, whereby the homogeneity of factor loadings was assessed. This was followed by scalar invariance testing, which interrogated the constancy of item intercepts across groups. The final evaluative tier, strict invariance, examined the uniformity of residual variances, therefore encompassing the most restrictive form of invariance testing.

Comparative model evaluations were conducted via absolute fit indexes, notably the Comparative Fit Index (CFI) and the Root Mean Square Error of Approximation (RMSEA), alongside the calculation of differential fit statistics (ΔCFI and ΔRMSEA) to assess relative model stability. According to the criteria posited by Chen (2007), invariance across models is substantiated when ΔCFI does not exceed 0.010 and ΔRMSEA remains below or equal to 0.015.

The empirical outcomes, as documented in Table 3, reveal marginal fluctuations only in fit statistics across the sequenced invariance tests, with ΔCFI ≤ 0.004 and ΔRMSEA ≤ 0.001. These findings robustly affirm that the measurement model fulfills all four thresholds of factorial invariance across gender groups.

**Table 3:** Invariance measures by sex.

Models	*X* ^2^	df	CFI	RMSEA	ΔCFI	ΔRMSEA
Sex (Women vs. Men)						
Configural	1368.26**	494	0.938	0.052	-	-
Metric	1409.06**	515	0.937	0.051	0.001	0.001
Scalar	1468.85**	536	0.934	0.051	0.003	0.000
Strict	1547.07**	560	0.930	0.052	0.004	−0.001

** *p* < 0.05. In this analysis, the participants’ gender variable is considered; df, degrees of freedom.

### Convergent and discriminant validity

Convergent and discriminant validity were examined through the correlational analysis of the constructs of emotional regulation and personality, respectively, employing the Emotional Regulation Questionnaire (ERQ) and the Mini-IPIP inventory grounded in the Five-Factor Model of Personality. The associations among variables were assessed via Pearson’s correlation coefficient, chosen for its robustness in evaluating the linear relationships between theoretically related yet distinct psychological dimensions.

The TMMS-24 scales are correlated with each other, with correlation ranges from 0.495 to 0.688, indicating medium to large (Cohen, 1992) effect sizes (see Table 4). Convergent and discriminant validity was assessed using the emotion regulation scales and the five-factor personality scale. Attention, clarity, and emotional repair are directly related to cognitive reappraisal, with correlation values ranging from 0.253 to 0.523, indicating small, medium, and large effect sizes. On the other hand, they show an inverse relationship with suppression, with *r* values ranging from −0.057 to −0.158, reflecting small effect sizes. The three TMMS-24 scales are also directly related to the five personality factors, with r values ranging from 0.180 to 0.499, corresponding to small and medium effect sizes. The results are presented in Table 4.

**Table 4:** Correlation of TMMS-24 scales with emotion regulation and personality.

Variables	M (SD)	*n*	1	2	3	4	5	6	7	8	9	10
1. Attention	24.12 (6.52)	1,315	--									
2. Clarity	23.49 (6.70)	1,315	0.648^**^	--								
3. Repair	24.58 (6.58)	1,315	0.495^**^	0.688^**^	--							
4. Reappraisal	28.11 (6.23)	1,315	0.253^**^	0.354^**^	0.523^**^	--						
5. Suppression	17.22 (4.61)	1,315	−0.153^**^	−0.158^**^	−0.057^*^	0.209^**^	--					
6. Extraversion	10.43 (3.40)	208	0.366^**^	0.402^**^	0.366^**^	0.250^**^	−0.206^**^	--				
7. Agreeableness	13.33 (3.57)	208	0.455^**^	0.454^**^	0.424^**^	0.312^**^	−0.173^**^	0.394^**^	--			
8. Conscientiousness	12.78 (3.48)	208	0.287^**^	0.359^**^	0.364^**^	0.226^**^	−0.144^**^	0.275^**^	0.400^**^	--		
9. Neuroticism***	11.26 (3.01)	208	0.180^**^	0.499^**^	0.495^**^	0.351^**^	−0.116	0.409^**^	0.414^**^	0.362^**^	--	
10. Openness to experience	13.45 (3.31)	208	0.269^**^	0.262^**^	0.355^**^	0.289^**^	−0.085	0.308^**^	0.417^**^	0.416^**^	0.329^**^	--

Variables	M (SD)	*n*	1	2	3	4	5	6	7	8	9	10
1. Attention	24.12 (6.52)	1,315	--	0.650^**^	0.545^**^	0.299^**^	−0.155^**^	0.267^**^	0.412^**^	0.311^**^	0.348^**^	0.307^**^
2. Clarity	23.49 (6.70)	1,315	0.663^**^	--	0.667^**^	0.355^**^	−0.225^**^	0.289^**^	0.485^**^	0.326^**^	0.420^**^	0.303^**^
3. Repair	24.58 (6.58)	1,315	0.504^**^	0.655^**^	--	0.537^**^	−0.123^**^	0.256^**^	0.437^**^	0.287^**^	0.287^**^	0.322^**^
4. Reappraisal	28.11 (6.23)	1,315	0.178^**^	0.335^**^	0.488^**^	--	0.138^**^	0.180^*^	0.343^**^	0.201^*^	0.337^**^	0.238^**^
5. Suppression	17.22 (4.61)	1,315	−0.151^**^	−0.091	0.002	0.313^**^	--	−0.337^**^	−0.284^**^	−0.207^*^	−0.239^**^	−0.136
6. Extraversion	10.43 (3.40)	208	0.325^*^	0.370^**^	0.300^*^	0.415^**^	0.126	--	0.401^**^	0.312^**^	0.426^**^	0.260^**^
7. Agreeableness	13.33 (3.57)	208	0.355^**^	0.429^**^	0.300^*^	0.286^*^	0.327^**^	0.495^**^	--	0.448^**^	0.442^**^	0.465^**^
8. Conscientiousness	12.78 (3.48)	208	0.220^*^	0.259^*^	0.373^**^	0.293^*^	0.075	0.183	0.301^*^	--	0.309^**^	0.439^**^
9. Neuroticism***	11.26 (3.01)	208	0.084	0.248	0.263^*^	0.386^**^	0.237	0.325^**^	0.491^**^	0.537^**^	--	0.313^**^
10. Openness to experience	13.45 (3.31)	208	0.264^*^	0.265^*^	0.341^**^	0.451^**^	0.098	0.460^**^	0.333^*^	0.341^**^	0.382^**^	--

*** Neuroticism refers to the positive version of the *N* factor, therefore it measures emotional stability; M, mean; SD, standard deviation. The data on the diagonal of the table are the correlations in the group of women, and below the diagonal are the correlations in the group of men.

** *p* < 0.01; * *p* < 0.05.

## Discussion and conclusion

The main objective of the study was to examine the psychometric properties of the Trait Meta-Mood Scale-24 (TMMS-24) in a sample of Peruvian university students. The results confirmed high reliability and internal consistency for the TMMS-24, with Cronbach’s alpha coefficients above 0.80 across the three subscales. These findings are consistent with those reported in the validation of the original version (Salovey et al., 1995), the Spanish adaptation (Fernández-Berrocal et al., 2004), as well as studies conducted in various sociocultural contexts.

Regarding test–retest reliability, this study, pioneering in the Peruvian context, found significant and substantial correlations, which reinforce the temporal stability of TMMS-24 scores in this population.

The three-dimensional factorial structure (Attention, Clarity, and Repair) of the TMMS-24 was confirmed through Confirmatory Factor Analysis (CFA), aligning with the original theoretical model and the conceptualization of emotional intelligence as the ability to perceive, understand, and regulate emotions (Fernández-Berrocal et al., 2004; Salovey et al., 1995). The construct validity of the Spanish version of the TMMS-24 for use in Peruvian university students is supported by the observed factor loadings, which ranged from 0.32 to 0.85. Similar findings have been documented in research with Polish adolescents (Cabello et al., 2025), Italians (Patti-Signorelli and de Romero-Díaz, 2023), Mexicans (Valdivia et al., 2015), Brazilians (Câmara et al., 2023), older adults (Delhom et al., 2017), young Argentinians (Gonzalez et al., 2021), and other studies involving Peruvian university students (Bueno-Cuadra et al., 2023).

Regarding gender differences, results indicated that men scored significantly higher on Emotional Clarity and Repair, while no significant differences were found in Emotional Attention. Some researchers have shown how women present a greater tendency to attend to their emotions and lower clarity and repair in comparison with men (Thayer et al., 2003), although these findings are not consistent and depend on variables such as age and cultural factors (Delhom et al., 2024; Fernández-Berrocal et al., 2004; Górriz et al., 2021; Salguero et al., 2010; Taramuel and Zapata, 2017). These variations suggest the potential influence of sociodemographic, developmental, and cultural factors on EI, highlighting the need for continued exploration of these differences in future research.

With respect to convergent and discriminant validity, results from the emotional regulation scales indicated a positive correlation between cognitive reappraisal and EI, while suppression was negatively associated. These findings align with Pastor et al. (2019), who found that cognitive reappraisal is associated with emotional attention and repair, whereas expressive suppression is negatively related to emotional clarity and repair on the TMMS-24. The positive correlation which was identified among the three dimensions of the TMMS-24 and the strategy of cognitive reappraisal provides empirical support for the hypothesis that heightened emotional competence facilitates the deployment of adaptive regulatory mechanisms—particularly, by the reframing of adverse experiences to attenuate their emotional impact. This association has been consistently substantiated in the literature, wherein cognitive reappraisal has been shown to mediate the positive relationship between emotional intelligence (EI) and psychological well-being, mitigating the detrimental effects of anxiety, depressive symptomatology, and academic stress (Barros and Sacau-Fontenla, 2021; Pastor et al., 2019; Shengyao et al., 2024). Indeed, Gross and John (2003) underscore that cognitive reappraisal is intrinsically linked to enhanced emotional regulation, increased emotional self-efficacy, and diminished negative affect—all of which constitute essential components of a functionally integrated model of EI.

Regarding the personality dimensions (Extraversion, Agreeableness, Conscientiousness, Neuroticism, and Openness), positive and significant correlations were found with the three TMMS-24 dimensions. These results are consistent with previous research that has shown relationships between perceived EI and various personality traits (Ghiabi and Besharat, 2011; Mayer et al., 2004; Saklofske et al., 2007; Salguero et al., 2010). The correlational patterns observed with the five major personality dimensions substantiate that, although emotional intelligence (EI) constitutes an autonomous psychological construct, perceived EI, nonetheless, portrays meaningful associations on top of relatively stable dispositional traits. To be specific, the findings reveal positive correlations between perceived EI and the traits of extraversion, agreeableness, conscientiousness, and willingness to the experience, suggesting that these personality factors are linked to more refined capacities for emotional recognition, understanding, and regulation both intra-and interpersonally. Much to the contrary, neuroticism, when reconceptualized as its inverse, emotional stability, demonstrates a negative correlation with perceived EI, indicating that higher emotional stability tends to align with greater emotional clarity and regulatory competence. This relationship corroborated by contemporary empirical work (Delhom et al., 2017; Mancini et al., 2024).

Regarding the concurrent validity of the instrument, it is particularly relevant to critically juxtapose these findings with recent Latin American research that has examined emotional intelligence using alternative psychometric scales. In the Colombian context, Arias-Gómez and Jiménez-Toro (2022), through the application of the EQ-i:S, identified an inverse relationship between EI and academic burnout, further reinforcing the construct’s protective function in higher education settings. These findings converge in affirming the consistent linkage between EI and key indicators of psychological well-being and academic adaptability. Thus, the consistency of the TMMS-24 findings backed up with this broader body of evidence strengthens the argument for its concurrent validity and affirms its appropriateness as a diagnostic measure within Latin American university populations.

The study has some limitations. For instance, the sample used consisted solely of university students, limiting the generalizability of the findings to other populations. Moreover, the cross-sectional design of the study prevents establishing causal relationships between the variables. Further studies could examine the validity of the TMMS-24 in different demographic groups, such as adults and older adults within the Peruvian population. It would also be advisable to investigate the relationship between the TMMS-24 and other relevant psychological variables in the Peruvian context, such as mental health, psychological well-being, academic performance, and social adjustment.

The ramifications derived from these findings are both contextually grounded and strategically significant for educational and mental health interventions within the Peruvian university system. The TMMS-24 emerges as a diagnostically valuable instrument for identifying students’ emotional competencies, therefore, enabling the implementation of targeted psychoeducational initiatives aimed at enhancing emotional regulation and psychological resilience. Accordingly, the integration of the TMMS-24 into student guidance and development frameworks offers a promising avenue for the design of emotionally attuned training programs, which may, in fact, foster improved mental health outcomes and bolster academic performance among university students in Peru.

In conclusion, the results of the present study provide strong evidence supporting the sound psychometric properties of the Spanish version of the TMMS-24 for use with Peruvian university students. These findings contribute to the literature on EI assessment in diverse cultural contexts and offer a valid and reliable tool for future psychological research, including cross-cultural studies and the evaluation of emotional education programs in Peru.

## Data Availability

The raw data supporting the conclusions of this article will be made available by the authors, without undue reservation.
